# NMDA Receptors Are Not Required for Pattern Completion During Associative Memory Recall

**DOI:** 10.1371/journal.pone.0019326

**Published:** 2011-04-29

**Authors:** Bing Mei, Fei Li, Yiran Gu, Zhenzhong Cui, Joe Z. Tsien

**Affiliations:** 1 Shanghai Institute of Brain Functional Genomics, Key Laboratory of Brain Functional Genomics, MOE and STCSM, East China Normal University, Shanghai, China; 2 Department of Developmental and Behavioral Pediatrics, Key Laboratory of Children's Environmental Health, MOE and STCSM, Shanghai Children's Medical Center, Jiao Tong University School of Medicine, Shanghai, China; 3 Banna Biomedical Research Institute, Xi Shuang Ban Na, Yunnan, China; 4 Brain and Behavior Discovery Institute and Department of Neurology, Georgia Health Sciences University, Augusta, Georgia, United States of America; University of Chicago, United States of America

## Abstract

Pattern completion, the ability to retrieve complete memories initiated by subsets of external cues, has been a major focus of many computation models. A previously study reports that such pattern completion requires NMDA receptors in the hippocampus. However, such a claim was derived from a non-inducible gene knockout experiment in which the NMDA receptors were absent throughout all stages of memory processes as well as animal's adult life. This raises the critical question regarding whether the previously described results were truly resulting from the requirement of the NMDA receptors in retrieval. Here, we have examined the role of the NMDA receptors in pattern completion via inducible knockout of NMDA receptors limited to the memory retrieval stage. By using two independent mouse lines, we found that inducible knockout mice, lacking NMDA receptor in either forebrain or hippocampus CA1 region at the time of memory retrieval, exhibited normal recall of associative spatial reference memory regardless of whether retrievals took place under full-cue or partial-cue conditions. Moreover, systemic antagonism of NMDA receptor during retention tests also had no effect on full-cue or partial-cue recall of spatial water maze memories. Thus, both genetic and pharmacological experiments collectively demonstrate that pattern completion during spatial associative memory recall does not require the NMDA receptor in the hippocampus or forebrain.

## Introduction

Memory retrieval is a rapid reconstructive process involving a recapitulation of the previously acquired information [1]–[3]. Quite often, memory retrieval occurs upon re-exposures to some, but not all, of the previously encountered cues or experiences. This ability to reconstruct and retrieve entire memory patterns from partial or degrade cued input is known as pattern completion. Currently, little is known about the actual molecular and cellular mechanisms underlying memory recall [4]–[7].

Since the NMDA receptor channel has a longer opening-duration, it has been speculated by computational biologists that the NMDA receptor might be a candidate molecule for initiating pattern completion within the auto-associative memory network during memory retrieval. In line with such a speculation, a previous study reports that CA3-specific NMDA receptor knockout mice exhibited performance deficits during the recall of spatial reference memory under the partial cue condition [8]. While this result, on its face value, seems to provide the only experimental evidence for the role of the NMDA receptor in pattern completion during associative memory recall, it carries a significant caveat because the gene knockout used in that study lacked inducible temporal controls, and as a result, the NMDA receptor was absent in all stages of memory processes (and in fact, most adulthood) [8]. Because it is well known that the NMDA receptor is required for learning, consolidation and storage [9], it is possible that the performance deficits observed in CA3-specific knockout mice under the partial-cue condition might have well reflected weak binding of various memory traces during the acquisition and consolidation stages due to the lack of the NMDA receptor during those stages. It is conceivable that weak memory traces formed during learning may not be obvious under the full cue recall conditions, but became detectable under the partial cue condition. This pattern completion deficit during recall may lead to a false interpretation that the NMDA receptor in the hippocampus is required for associative memory recall [8].

To re-examine the role of NMDA receptors in pattern completions during memory retrieval, we set out to apply inducible and region-specific gene knockout methods that would allow us to restrict the NMDA receptor knockout to the recall phase. We generated two independent lines of mice in which inducible knockout of the NR1 gene can be temporally restricted to the memory retrieval stage and spatially limited to either the hippocampal CA1 region (iCA1-KO) or the entire hippocampus regions (CA1, CA3, and dentate gyrus) and cortex (iFB-KO). In our experiments, the animals would acquire and consolidate memory normally in the presence of the NMDA receptor (before inducible knockout), but memory retrieval occurs in the absence of the NMDA receptor (after inducible knockout). With this temporally controlled method, we find that the mice lacking hippocampal and cortical NMDA receptors at the time of memory recall exhibited normal pattern completion. Thus, our genetic analyses suggest that the NMDA receptor is not required for pattern completion during associative memory recall.

## Results

We produced two strains of inducible and region-specific NMDA receptor knockout mice; namely, inducible and CA1-specific NR1 knockout mice (iCA1-KO) [10] and inducible and forebrain-specific NR1 knockout mice [11]–[12]. We used the identical spatial reference memory protocol that was used in the previous study. We first subjected the iCA1-KO mice and their littermates to the hidden-platform water maze task to assess their ability to form a spatial reference memory. It is known that the hippocampal CA1 NMDA receptor is crucial for the acquisition as well as the consolidation of this form of spatial reference memory [9], [10], [13], [14]. Thus, to avoid any disruption in learning and consolidation, we feed the mice with normal food (no doxycycline, thus, with normal NMDA receptor function in CA1) and trained both iCA1-KO and their control littermates in the hidden-platform water maze task with four prominent, distal visual cues hung on the surrounding black curtain. The training is consisted of four trials per day, with one hour inter-trial-intervals. The entry of mice to the pool was randomized so that each mouse would enter the pool from each of the four quadrants in the one-day four trials.

**Figure 1:**
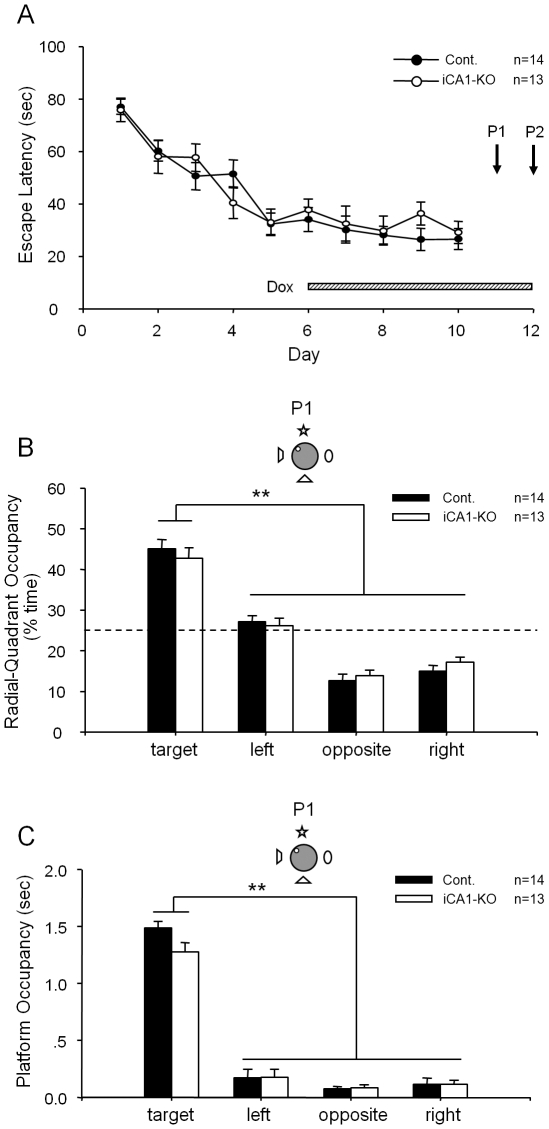
Normal formation of spatial reference memory in iCA1-KO and control mice as measured by the hidden-platform water maze. (A) Normal learning of spatial reference memory in iCA-KO (n = 13) and their control littermates (n = 14) as measured by escape latency. The shaded bar illustrates the five-day-Dox-feeding duration so that memory retrieval on Day 11 and 12 occurred in the absence of the NMDA receptor. Two probe tests (P1 and P2) were conducted. (B) Normal retrieval in iCA-KO mice under full-cue condition (probe test #1, or P1) in the absence of CA1 NMDA receptors. Both types of mice exhibited strong preference toward the target quadrant during their 90 seconds search (P<0.01). (C) The measurement of platform occupancy further shows that both types of mice crossed the phantom platform location more significantly than the similar locations in other quadrants (P<0.01), suggesting normal pattern completion in the absence of CA1 NMDA receptors. Data were calculated as Mean ± SEM. **Indicates a significant between group difference for a given time point (**p<0.01).

We have found that both the iCA1-KO mice with the functional CA1-NMDA receptors and their control littermate learned the task well. As shown by the escape latency, both groups of mice gradually reduced the amount of time to find the hidden platform over the course of training, and there was no difference in the acquisition rate of spatial learning among the genotypes (Figure 1A), indicating that iCA1-KO mice exhibited normal learning and consolidation in comparison to that of the control littermates. Also, no significant differences were observed between the control and iCA1-KO mice in the path length, average swimming velocity, or wall-hugging time (data not shown).

To confirm the spatial learning of this reference task, we subjected both groups of mice to a full-cue probe trial on the day after the completion of 10 day training sessions (probe test #1, P1). During this test, the hidden platform was removed, and the mice were allowed to search in the pool for 90 sec. Their spatial memories were assessed by monitoring the relative radial-quadrant occupancy. If the animals do not learn the location of the fixed hidden platform, they would not show any preference of their search toward the target quadrant. On the other hand, if the animals learned the location of the hidden platform, they would concentrate their search in the target quadrant in which the platform was previously located. Since it is known that it takes about 5 days of dox-treatment to switch off the CA1 NMDA receptor function, we switched the regular food with dox-containing food pellets in the evening of training day 6 so that by the P1 test, the NMDA receptor in the CA1 region of the iCA1-KO mice would be completely knocked out. As a control, we also treated the littermate control group with the same dox-containing food pellets. Interestingly, on P1 test with the presence of all four cues on the curtain wall, both groups spent significantly more time in the target quadrant than any other non-target quadrants (Figure 1B). In addition, the measurement of platform crossing-time also showed no difference between the iCA1-KO and control groups (Figure 1C). Thus, our results suggest that inducible knockout of CA1 NMDA receptors at the time of memory recall had no effect on the retrieval of spatial reference memories under the full cue condition.

**Figure 2:**
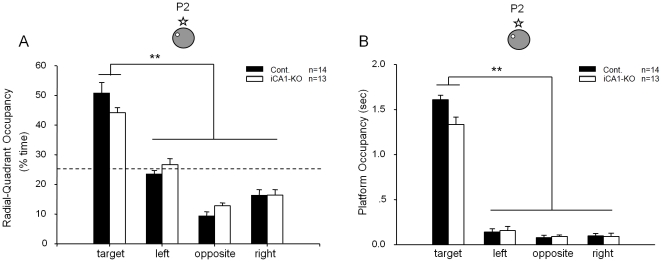
Normal memory recall by iCA1-KO mice. (A) Normal retrieval in iCA-KO mice under partial-cue condition (probe test #2, or P2) as measured by quadrant occupancy. iCA-KO mice, n = 13; control littermates, n = 14. (B) The measurement of platform occupancy further shows that both types of mice crossed the phantom platform location more significantly than the similar locations in other quadrants, thereby confirming the normal pattern completion during memory recall by iCA1-KO mice even in the absence of CA1 NMDA receptors. The location of the hidden-platform and one remaining cue hung on the surrounding black curtain wall are illustrated.

To investigate the role of hippocampal NMDA receptors in pattern completion under the partial cue condition, the following day we removed three of the four distal visual cues from the surrounding curtain and re-examined spatial memory recall by these mice by subjecting them to the second probe test (P2). In this partial cue probe trial, both the iCA1-KO and control mice, again, spent a significant amount of time in the target quadrant than other quadrants (Figure 2A). Moreover, both iCA1-KO and control groups searched the phantom platform location (indicated by the platform crossing-time) as much as they did in the full-cue condition (Figure 2B). Therefore, those results suggest that the pattern completion occurred normally despite the lack of CA1 NMDA receptor during associative spatial memory recall.

**Figure 3:**
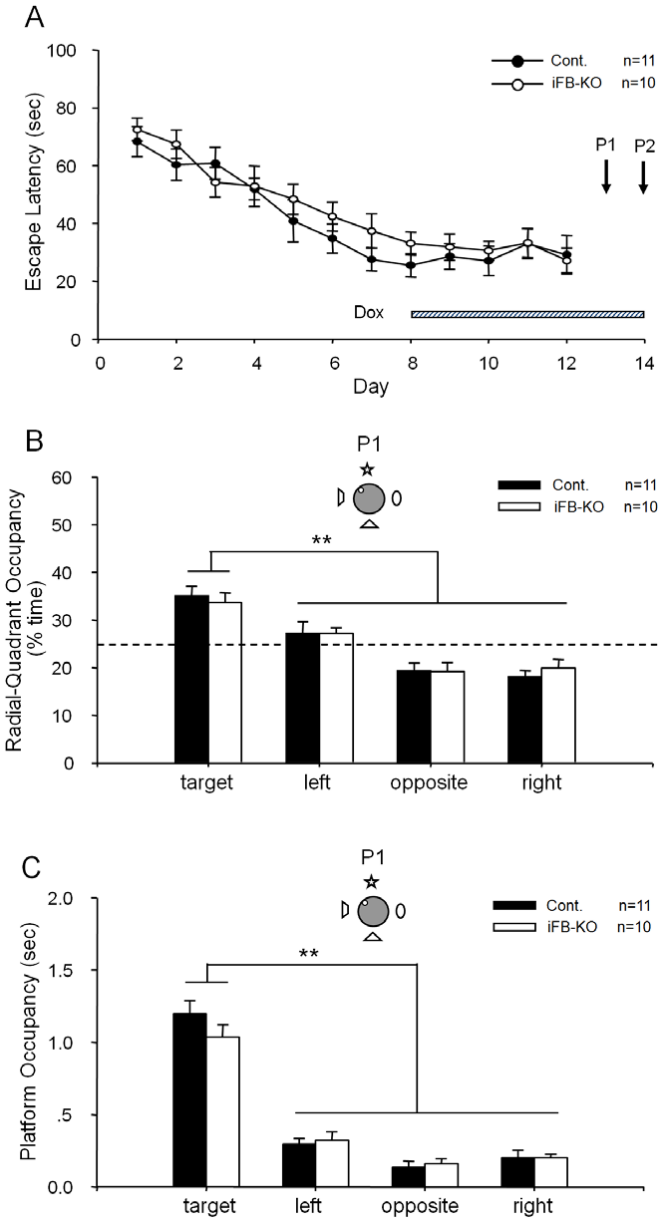
Formation of spatial reference memory in iFB-KO and control mice as measured by the hidden-platform water maze. (A) Normal learning of spatial reference memory in iFB-KO (n = 10) and their control littermates (n = 11) as measured by escape latency. The shaded bar illustrates the five-day-Dox-feeding duration so that memory retrieval on the probe trial #1 and #2 would occur in the absence of the NMDA receptor. Two probe tests (P1 and P2) were conducted. The Cre-62 line was used for achieving forebrain-specific NR1 knockout [11]. (B). Normal retrieval in iFB-KO mice under full-cue condition (probe test #1, or P1) in the absence of forebrain NMDA receptors. Both types of mice exhibited strong preference toward the target quadrant during their 90 seconds search (P<0.01). (C) The measurement of platform occupancy further shows that both types of mice crossed the phantom platform location more significantly than the similar locations in other three quadrants (P<0.01), suggesting the normal memory retrieval (full cue condition) by iFB-KO mice in the absence of the NMDA receptors in the forebrain regions including the cortex and all subregions of the hippocampus. The location of the hidden-platform and four visual cues on the surrounding black curtain wall are illustrated.

Lack of apparent requirement of CA1 NMDA receptors for the pattern completion has led us to entertain the following two alternative scenarios: 1) CA1 might be just one of many regions involved in retrieving memory, the knockout of CA1 NMDA receptor is insufficient to see the pattern completion deficit. 2) The NMDA receptor is not required at all for associative memory recall. To distinguish those two scenarios, we further produced inducible and forebrain-specific NR1 knockout mice (iFB-KO) in which the NMDA receptor can be temporally deleted in the entire hippocampus (CA1, CA3, and dentate gyrus) and the neocortex upon feeding of dox–containing food [11], [12]. Using the same 4-trials-per-day training protocol and training environment, we first trained a group of iFB-KO and control littermates in the hidden platform water maze. Since those iFB-KO mice were fed with normal food (no dox), the mice acquired spatial reference memory about the fixed location of the hidden platform with functional NMDA receptors in their cortical and hippocampus. As expected, those iFB-KO mice exhibited normal learning-curve in comparison to their control littermates (Figure 3A). Both groups of mice reduced the amount of time in locating the hidden platform over daily training sessions. The formation of spatial reference memory is further demonstrated by the probe tests. In the first full-cue probe test (P1), those mice showed significant preference in term of spending more time in the target quadrant during the 90-second probe trial session (Figure 3B). Moreover, iFB-KO mice also had a similar amount of platform crossing-time in comparison to that of the control mice (Figure 3C). Since we treated both types of the mice with dox food pellets for 5 days before the first probe test, the iFB-KO mice should have no functional NMDA receptor in the forebrain regions at the time of memory recall. Our previous experiments have shown that it takes 5-days of dox treatment to completely suppress NR1 gene expression in inducible NR1 knockout mice [10]. Therefore, by starting the dox treatment on day 8 and testing the retention on the first day of NR1 complete knockout (on P1) can greatly minimize any potential effects on consolidation [10]. The normal retrieval performance in this set of experiments described in Figure 3 strongly suggest that temporally restricted forebrain NMDA receptor knockout prior to retention test had no effect on memory recall under the full cue condition.

**Figure 4:**
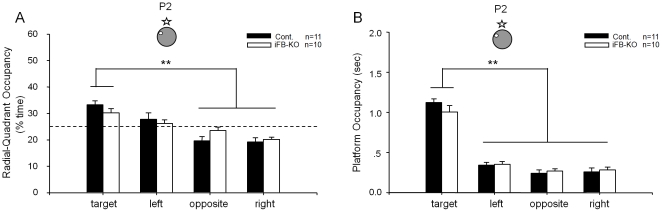
Normal memory recall by iFB-KO mice. (A) Normal memory retrieval under partial-cue condition (probe test #2, or P2) as measured by quadrant occupancy by iFB-KO mice in the absence of the NMDA receptor in the forebrain regions such as the cortex and all subregions of the hippocampus. iFB-KO mice, n = 10; control littermates, n = 11. (B) The measurement of platform occupancy further shows that both iFB-KO and their control littermates spent more time crossing the phantom platform location than crossing the similar locations in other quadrants, thereby further showing that the normal pattern completion during memory recall by iFB-KO mice took place even in the absence of forebrain NMDA receptors. The location of the hidden-platform and the remaining cue hung on the surrounding black curtain wall are illustrated.

To determine whether the forebrain NMDA receptor is necessary for pattern completion under the partial cue condition, the next day we conducted the second probe test (P2) in which three of the four distal cues were removed. During this partial cue probe trial, both the iFB-KO and control mice continued to concentrate their search time in the target quadrant than other quadrants (Figure 4A). There is a significant difference between target radial-quadrant occupancy vs. the mean of radial-quadrant occupancy in the other three quadrants (see Figure S1, p<0.01). More importantly, the platform crossing measurement showed that iFB-KO and control groups searched the phantom platform location as much as they did in the full-cue condition (Figure 4B), also suggesting no significant extinction effect from the probe test #1. Our measurement of crossing-time further revealed no statistically significant difference between the iFB-KO-KO and control groups. Thus, these experiments suggest that iFB-KO mice lacking cortical and entire hippocampal NMDA receptors at the time of memory recall still retrieved spatial reference memories normally even under the partial cue condition.

**Figure 5:**
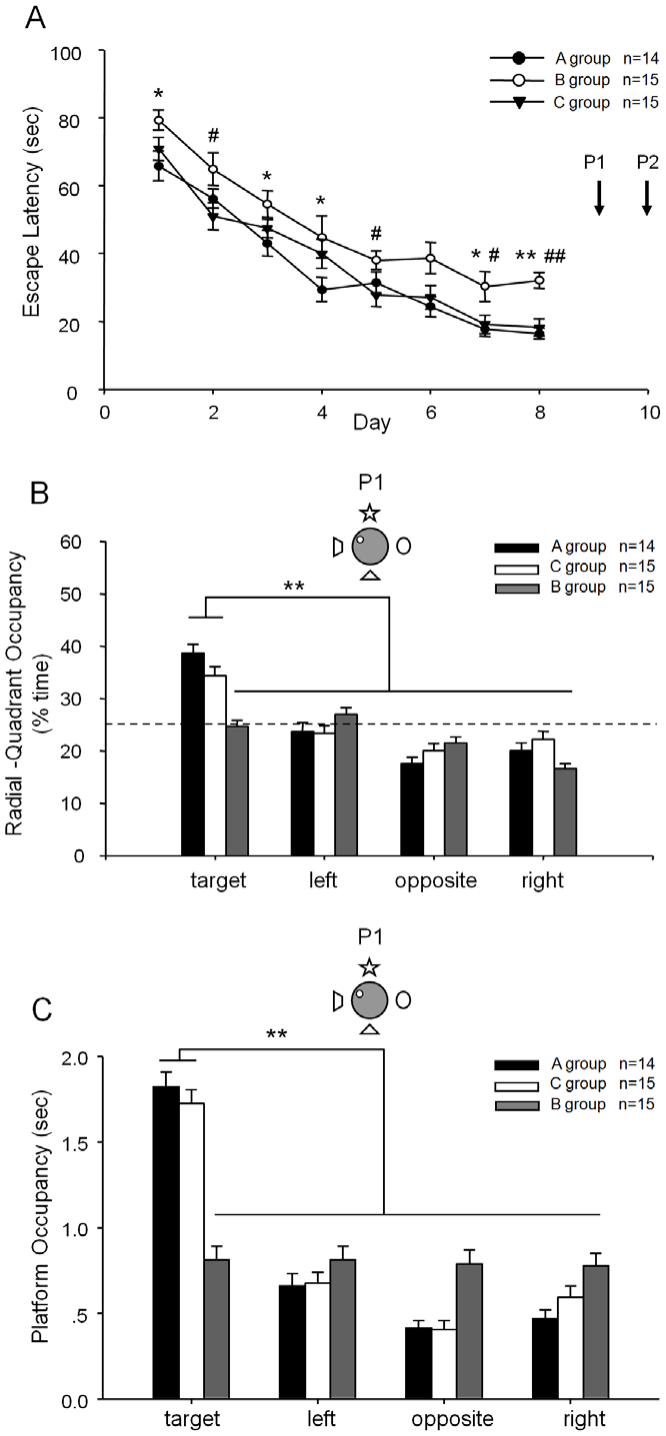
Pharmacological blockade of NMDA receptor has different effect on formation of spatial reference memory. Three groups of wild type mice (B6/CBAF1, age 4–6 months) were used. Group A received i.p. injection of saline 1.5 hour before experiment for both training and probe test; Group B received i.p. injection CPP (10 mg/kg) 1.5 hour before experiment for training but received saline injection during the probe tests (NMDA receptor antagonism during learning but not during recall). Group C received i.p. injection of saline 1.5 hour before experiment for training but CPP at the time of the probe tests (NMDA receptor antagonism only during recall). (A) Mice received CPP during training sessions showed impairment at learning (*, Group A vs. Group B, #, Group C vs. Group B, */# p<0.05; **/##, p<0.01). (B) Normal memory retrieval under full cue condition as measured by target quadrant occupancy. It confirmed that group B received CPP during training session failed to show any preference to the target quadrant, suggesting the effectiveness of CPP in blocking NMDA receptor function. However, the mice from the Group C which received CPP treatment at the time of recall still exhibited strong preference to the target quadrant under the full cue retrieval condition. (C) Normal memory recall condition under full cues condition was further confirmed by the measurement of platform crossing-time. ** p<0.01.

To further examine the role of the NMDA receptor in pattern completion, we used systemic injection of the NMDA receptor pharmacological antagonist, 3-[(R)-2-carboxypiperazin-4-yl]-propyl-1-phophonic acid (CPP) at the dose of 10 mg/kg [15]. This pharmacological approach may allow us to complement the above genetic methods. We used three groups of wild-type mice: Group A which received saline injection, Group B which received CPP treatment during all the training sessions, and Group C which received CPP treatment at the time of recall test. As expected, the mice received CPP injection (Group B) at the learning phase exhibited a slower learning curve. There is a significant difference between Group B and the other groups without CPP treatment (Fig. 5A). The measurement of target quadrant occupancy and platform crossing-time further confirmed that Group B performed at the chance level (Fig. 5B and 5C). Furthermore, Group C, which received CPP injection 1 hour before the retention tests, exhibited normal memory retrieval under the full cue condition of the first probe test (P1) (Fig. 5B and 5C). These results indicate the CPP blockade impaired learning but not memory recall.

**Figure 6:**
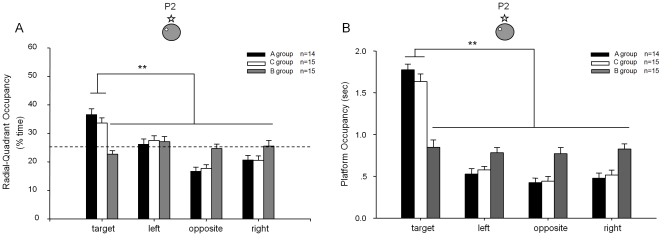
Normal pattern completion partial-cue condition was also confirmed by Pharmacological blockade of NMDA receptor. (A) Under the partial cue condition, mice receiving CPP at the time of recall still spent more time in the target quadrant. (B) Normal memory recall condition under partial cue was further confirmed by the measurement of platform crossing-time, thereby further showing that the normal pattern completion during memory recall. The location of the hidden-platform and the remaining cue hung on the surrounding black curtain wall are illustrated. ** p<0.01.

On the following day, we subjected all mice to the second probe tests (P2) under partial cue condition. As measured by both target quadrant occupancy and platform crossing-time, Group A showed normal recall (Fig. 6A and 6B). Not surprisingly, Group B remained impaired under this partial cue recall test, as they were impaired in learning the platform location during training (Fig. 6A and 6B). Interestingly, Group C, receiving CPP injection prior to the partial cue recall test, also had normal retrieval ability as judged by the stronger preference to the target quadrant as well as the equal time of crossing in comparison to the control mice (Fig. 6A and 6B).

## Discussion

Our inducible knockout of cortical and hippocampal NMDA receptors restricted to the memory retrieval phase had no effects on pattern completion during associative memory recall. In addition, the NMDA receptor antagonist CPP experiments also fail to reveal any impact on partial cue retrieval. So, what are the factors that might explain the apparent discrepancy between our current study and the previous CA3-knockut study? One potential argument might be that CA3 is a crucial region known to have extensive recurrent connections, whereas CA1 has only limited recurrent connections and also known to be activated during recall. Thus, the CA3 might be better positioned to play a role in auto-associative pattern completion than the CA1 [16]. As such, the knockout of the CA1 would be less likely to affect pattern completion than the knockout in CA3. In other words, due to distinct circuitry properties in memory processes, CA1 NMDA receptor is mostly involved in learning and consolidation, whereas, CA3 may be preferential for pattern completion during recall. Indeed, our previous work showed that the mice with inducible NR1 knockout in the CA1 region during acquisition resulted in impaired retention performance in the full cue condition [10]. In comparison, the mice lacking NMDA receptor in the CA3 were not impaired in the full cue condition [8]. This indicates that the NMDA receptors in the CA1 and CA3 may engage in different aspects of memory processing. It is possible that disabling of NMDA receptors in CA1 region would not produce any observable impairment on pattern completion during partial cued recall.

To examine this issue, we thus further applied inducible- and forebrain region-specific NMDA knockout method. In the forebrain-specific knockout mice, the inducible deletion of NMDA receptor in the forebrain regions includes 98% of pyramidal cells in CA1 and 90% of CA3 cells, and 95% of granule cells in the dentate gyrus of the hippocampus as well as 62% of cells in the neocortex [11]. These inducible CA1-specific or forebrain-specific NMDA knockout mice, once fed with doxycycline to disable NMDA receptor function, produced deficits in memory formation, consolidation, and storage [10], [11]. If the NMDA receptor (especially in CA3) were to play an essential role in pattern completion during memory retrieval, one should expect the forebrain knockout mice to exhibit much more profound memory retrieval deficits than that of CA3-specific NMDA knockout mice. However, our present experiments clearly show that the forebrain-specific knockout mice exhibited normal memory retrieval performance under either full-cue or partial-cue condition. This lack of effect on memory retrieval is in stark contrast to the dramatic and detrimental effects of the NMDA receptor knockout on learning, consolidation, and storage [10]–[13], [17], [18]. Although it can be argued that 90% of NR1 knockout in CA3 cells of the forebrain knockout mice may still leave residual amount of cells for pattern completion during recall, the systemic blockade of NMDA receptor function by CPP also did not affect partial cue-initiated pattern completion. It should be mentioned that this form of hidden-platform spatial water maze task is very sensitive to NMDA receptor deletion as we have shown previously in the CA1 pyramidal cell-specific NR knockout mice [13]. Moreover, we have also done the similar experiment on inducible iCA1-KO mice [10]. By acutely feeding the iCA1-KO mice with doxy (starting 5 days before the water maze training), we reported that doxy-treated iCA1-KO mice exhibited longer escape latency than doxy-treated control or vehicle-treated iCA1-KO mice [10]. The learning deficits in doxy-treated iCA1-KO mice were further confirmed in the transfer test [10]. Thus, the evidence for hippocampal NMDA receptor in learning is quite clear and highly consistent with our current CPP experiments. Collectively, these experiments have led us to conclude that the NMDA receptor is critical for learning/consolidation, but not essential for pattern completion during associative memory recall.

Our stringent genetic and pharmacological analyses are also consistent with human and animal studies showing that NMDA receptor antagonists generally spare memory retrieval [19]–[25]. Increasing amount of evidence indicates that retrieval may engage other types of neurotransmitter systems [26]–[30]. For example, we have recently shown that imbalance in dopamine resulting from heterozygous knockout of the dopamine transporter gene causes significant impairment in partial cue-initiated spatial memory recall [31].

A previous study reports that reversible inactivation of mossy-fibers, the input to CA3, affected spatial encoding, but not retrieval of spatial memory [32]. This adds to the literature that the hippocampus is critically involved in learning and episodic memory pattern consolidation [10], [33], whereas the same lesion of the dorsal hippocampus does not seem to produce significant impairment in retrieval of spatial or contextual memories [25], [34].

The second explanation, which is more likely, is that CA3-NR1 knockout occurred in all stages of memory processes (in fact throughout most of their adulthood), thereby the performance deficit described in the partial-cue condition might actually be a result of weak learning and consolidation of multiple memory traces representing external cues and the spatial task because extensive evidence suggests the crucial role of the NMDA receptor in memory acquisition, consolidation, and storage [9]–[12], [35], [36]. As such, this incomplete binding/consolidation of memory traces in CA3 knockout mice during learning and consolidation may only manifest itself under the partial-cue recall condition, but not under the full-cue condition because sensory inputs from full cues can compensate such weak binding of memory traces. This could lead to the misinterpretation of such learning/consolidation deficits as retrieval deficits.

The third possibility is that the reported partial-cue retrieval deficit in CA3 knockouts may be due to the compounded expression of a huge 120-kb-long bacterial artificial chromosome (BAC) used for the generation of CA3-spceific Cre line. This BAC fragment contains a part of the genomic sequence of mouse kainate receptor 1 subunit as well as other unknown sequences (e.g. regulatory RNAs). While Cre line seemed to be normal in its learning behaviors, it remains possible that once the Cre line was crossed into floxed NR1 line it may produce a compounded effect that may negatively impact memory recall. It is not clear whether separate DNA fragments for Cre transgene and 120-kb-BAC used during the pronucleus injection may increase the chance in disrupting unknown gene(s) or sequences at their integration sites, independent of the NMDA receptor function. These two types of integration-mediated complications or ectopic expression of transgene can, and should be, stringently tested by producing inducible and CA3–specific knockout mice since the phenotypes should be tightly correlated with dox-induced knockout period. The use of inducible knockout mice can resolve this type of concerns on potential ectopic expression of transgene since the phenotypes can be directly compared in two groups of the same strain of inducible knockout mice with one group treated with dox and another group without dox treatment. If the inducible knockout mice with dox treatment still exhibited normal performance, it is quite reasonable to interpret that ectopic integration or expression of the transgene does not have a direct role in producing a deficit. Our previous experiments suggest that transgenic overexpression of NR1-GFP can rescue the NR1 knockout state in the inducible mutant mice prior to dox treatment, whereas dox-treatment suppressed the NR1-GFP, thereby reverting to the knockout states in both physiological and behavioral terms [10]. Interestingly, our observation here that inducible knockout of the forebrain NMDA receptor at memory recall had no significant effect on pattern retrieval (using the same behavioral protocols) clearly contrasts with the conclusion drawn from non-inducible CA3 NMDA receptor knockout mice study. Our both pharmacological and inducible genetic experiments suggest that such pattern completion deficit, if any, is unlikely due to the direct effect of the NMDA receptor on memory recall. On the other hand, if inducible knockout of the NMDA receptor at memory recall did not produce any impairment, it is reasonable to interpret that any ectopic expression of the NR1-GFP gene or Cre transgene does not seem to interfere with recall (as in the present case) but can rescue NMDAR deficits occurred at other memory stages [10]–[12].

Of course, it might be equally possible that the originally reported minor pattern completion deficit in CA3-NR1 knockout mice may have resulted from behavioral variations during those particular sets of experiments (it is noted that an unusually large group of control mice were used in comparison with a much smaller group of mutant mice). It would be important to replicate the original finding with a matched number of mice. Resolving the role of the NMDA receptors on distinct stage of memory processes is critical for guiding computational models that have so often relied on the idea of NMDA receptors for achieving CA3 recurrent pattern completion. An emerging study point to the dopamine as a key regulator in pattern completion [31], although further studies are needed to identify the loci of such action.

In conclusion, our present experiments from both inducible knockout mice and pharmacological experiments demonstrate that the NMDA receptor in the cortex and hippocampus is not necessary for the pattern completion during associative memory recall in the spatial water maze task.

## Materials and Methods

### Ethic statements

All animal work described in the study were carried out in accordance with the guidelines laid down by the National Institutes of Health in the US regarding the care and use of animals for experimental procedures, and was approved by the Institutional Animal Care and Use Committee of the Georgia Health Sciences University (GHSU Approval AUP number: BR07-11-001).

### Production and genotyping of inducible knockout mice

Construction of conditional knockout mice was the same as described previously for inducible and CA1-specific NR1 knockout mice (iCA1-KO) [10] and for inducible and forebrain-specific NR1 knockout mice (iFB-KO) [11], [12]. All our transgenic mice were produced on BCF hybrid background (B6×CBAF1) [10] in the GHSU Animal facility, and the original floxed NR1 heterozygous, produced from across between 129 and B6 inbred [13], has been also crossed to the BCF hybrid strain for at least 20 generations. The inducible knockout mice are homozygous for the floxed-*NR1* gene and heterozygous for the CaMKII-*Cre* transgene (T29.1-Cre line for producing iCA1-KO, 62-Cre line for producing iFB-KO), the *NR1-GFP* transgene under control of the tet-O promoter, and the tetracycline transactivator (*tTA*) transgene, which is driven by the β-actin promoter and contains a floxed stop sequence (*fNR1*/*fNR1*, *Cre*/+, *tTA*/+, and *NR1-GFP*/+) [10], [11], [12]. The littermates lacking the Cre gene (*fNR1*/*fNR1*, *tTA*/+, *NR1-GFP*/+; or *fNR1*/+, *tTA*/+, *NR1-GFP*/+) were used as control mice. For our experiments, both male and female mice were equally used at the ratio about 50∶50. For genotyping, southern blot method was used to detect the floxed *NR1* gene and the protocol is the same as described [11], [17], [37]. About 10 µg-purified tail DNA were digested by EcoR I, fractionated by electrophoresis on 0.7% agarose gels and transferred onto Zeta-probe GT membranes (BioRad). A 1.2 kb DNA fragment of 3′ NR1 gene probe was labeled by α-32P-dCTP and hybridized to the GT membranes. For PCR detection of the *Cre*, *tTA*, and *NR1-GFP* transgenes, approximately 0.5 to1 µg of mouse tail DNA was amplified in PT100 thermal cycler using the programs as follows: 1 minute, 94°C; 45 sec, 55°C; and 1 min, 55°C for 35 cycles. The primers for *Cre* detection is 5′-AGA TGT TCG CGA TTA TC and 5′- AGC TAC ACC AGA GAC GG; for *tTA* detection is 5′- CAA TTA CGG GTC TAC CAT and 5′-GGT TCC TTC ACA AAG ATC CTC; and for *NR1-GFP* detection is 5′- GGT AGA GCA GAG CCC GAC CCT and 5′-GTA TCT GGA AAA GCA CTG respectively. The size of specific PCR products for *Cre* is 490 bp, 450 bp for *tTA*, and *NR1-GFP* 400 bp for NR1-GFP.

### Spatial reference memory tests

All mice were maintained under the standard condition (23.1°C, 50.5% humility) in the GHSU animal facility. All experiments were conducted in a soundproofed and specialized behavior room. All experimenters were blind to the genotype of the individual animal. The spatial reference memory test was the hidden-platform water maze. To ensure the CA1-specificity, the age of iCA1-KO mice by the time the retrieval experiments were completed was no larger than 9 weeks [13], [17]. The age of iFB-KO mice were between 4–8 months, which we have shown that Cre-62 line can achieve robust Cre/loxP recombination in the forebrain regions such as the cortex and all regions of the hippocampus (CA1, CA2, CA3, and dentate gyrus) [11], [12]. We used the same protocol as described previously [8] and the training consisted of four trials per day, with one hour inter-trial-interval. The movement of mice was tracked by a video camera. The escape latency to the platform, quadrant occupancy and platform crossing-time were all recorded and analyzed. The pool has the diameter of 118 cm and the platform is 9.5 cm in diameter. Data were calculated as Mean ± SEM. Two probe tests were performed. The first probe test (P1) conducted at the end of the last session under the full-cue condition and followed by the second probe test (P2) next day under the partial-cue condition (by removing three of the four visual cues hung on the black curtain wall). During the probe tests, the platform was removed and the mice were allowed to swim in the pool for 90 sec. The time spent in each quadrant was recorded. A One-way ANOVA and post hoc Dunnett's test were used to determine genotype effects.

For knocking out NMDA receptor function, we fed the mice with food pellets containing dox at 6 mg/g for five days because our previous studies showed it took five days for the pre-made NR1 protein to be completely degraded at synapses [10], [11]. Mice showed no preference or dislike for the dox containing food and consumed approximately the same amount in comparison to the regular food pellets. For the NMDA receptor antagonist study, we used 3-[(R)-2-carboxypiperazin-4-yl]-propyl-1-phophonic acid (CPP) at the dose of 10 mg/kg.

### Data analysis

To account for intra-animal correlations between repeated measurements, linear mixed models were employed to estimate the behavioral performance in the Morris water maze. The Tukey–Kramer method was used to determine the significance of those behavioral measurements between iCA1-KO (or iFB-KO, or CPP-injection mice) and their control littermates. A One-way ANOVA and post hoc Dunnett's test were used to determine genotype effects. Continuous variables are presented as the mean and standard error of the mean (SEM). Data were analyzed using SPSS version 13.0 (SPSS Inc., Chicago, IL). Differences were considered significant when P<0.05.

## Supporting Information

Figure S1 **Normal recall under partial cue condition in iFB-KO mice as measured by radial quadrant occupancy.**
(TIF)
