# Supplementary figures and images for: NMDA Receptors Are Not Required for Pattern Completion During Associative Memory Recall
Source: PLoS One. 2011 Apr 29;6(4):e19326. doi: 10.1371/journal.pone.0019326 (PMC3084823; doi:10.1371/journal.pone.0019326)

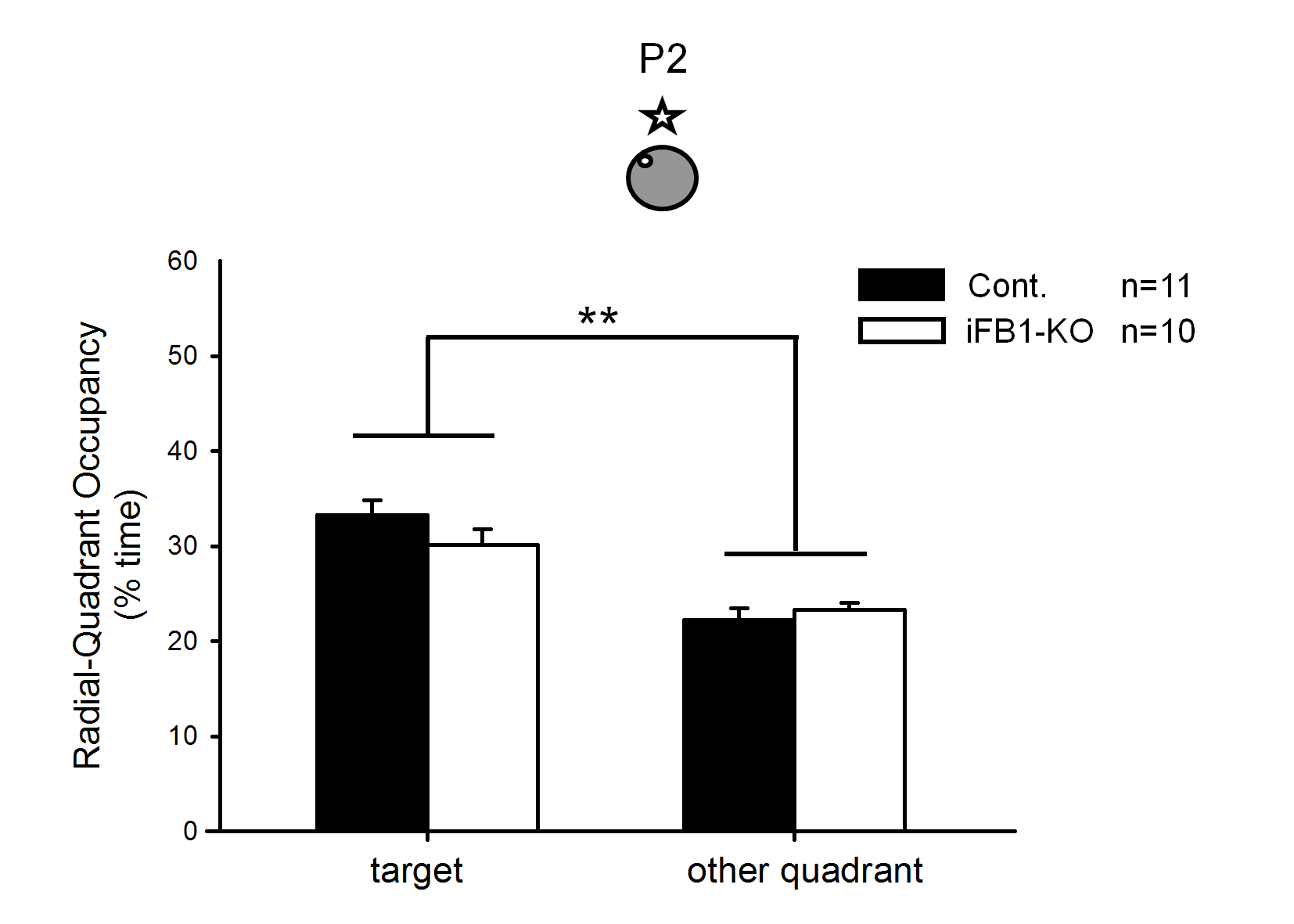

Supplement: Figure S1 — Normal recall under partial cue condition in iFB-KO mice as measured by radial quadrant occupancy. (TIF) [file pone.0019326.s001.tif]
